# Influence of Steroid Hormone Signaling on Life Span Control by *Caenorhabditis elegans* Insulin-Like Signaling

**DOI:** 10.1534/g3.112.005116

**Published:** 2013-05-01

**Authors:** Kathleen J. Dumas, Chunfang Guo, Hung-Jen Shih, Patrick J. Hu

**Affiliations:** * Graduate Program in Cellular and Molecular Biology; † Life Sciences Institute, University of Michigan, Ann Arbor, Michigan 48109; ‡ Departments of Internal Medicine and Cell and Developmental Biology, University of Michigan Medical School, Ann Arbor, Michigan 48109

**Keywords:** *Caenorhabditis elegans*, steroid hormones, insulin signaling, aging, longevity

## Abstract

Sterol-sensing nuclear receptors and insulin-like growth factor signaling play evolutionarily conserved roles in the control of aging. In the nematode *Caenorhabditis elegans*, bile acid-like steroid hormones known as dafachronic acids (DAs) influence longevity by binding to and regulating the activity of the conserved nuclear receptor DAF-12, and the insulin receptor (InsR) ortholog DAF-2 controls life span by inhibiting the FoxO transcription factor DAF-16. How the DA/DAF-12 pathway interacts with DAF-2/InsR signaling to control life span is poorly understood. Here we specifically investigated the roles of liganded and unliganded DAF-12 in life span control in the context of reduced DAF-2/InsR signaling. In animals with reduced *daf-2/InsR* activity, mutations that either reduce DA biosynthesis or fully abrogate DAF-12 activity shorten life span, suggesting that liganded DAF-12 promotes longevity. In animals with reduced DAF-2/InsR activity induced by *daf-2/InsR* RNAi, both liganded and unliganded DAF-12 promote longevity. However, in *daf-2/InsR* mutants, liganded and unliganded DAF-12 act in opposition to control life span. Thus, multiple DAF-12 activities influence life span in distinct ways in contexts of reduced DAF-2/InsR signaling. Our findings establish new roles for a conserved steroid signaling pathway in life span control and elucidate interactions among DA biosynthetic pathways, DAF-12, and DAF-2/InsR signaling in aging.

Steroid hormones have critical functions in development and maintenance of homeostasis throughout metazoan phylogeny. They exert their effects largely by binding to and regulating the activity of transcription factors of the nuclear receptor superfamily (Wollam and Antebi 2011). In the nematode *Caenorhabditis elegans*, bile acid-like steroid hormones known as dafachronic acids (DAs) are nuclear receptor ligands that control development and life span by binding to and regulating the activity of the nuclear receptor DAF-12 (Motola *et al.* 2006). Two structurally related DAs, Δ^4^- and Δ^7^-DA, differ in potency but appear to have similar functions in regulating larval development (Sharma *et al.* 2009).

Genetic analyses and rescue experiments with presumed DA biosynthetic intermediates are consistent with a model whereby Δ^4^- and Δ^7^-DA are synthesized from cholesterol via distinct pathways (Figure 1A) (Wollam *et al.* 2012). The Rieske oxygenase family member DAF-36 catalyzes the first step of Δ^7^-DA biosynthesis by synthesizing 7-dehydrocholesterol [7-DHC; (Rottiers *et al.* 2006; Wollam *et al.* 2011; Yoshiyama-Yanagawa *et al.* 2011)]. 7-DHC is thought to be converted into lathosterol, the 3-OH group of which is subsequently oxidized by the 3-hydroxysteroid dehydrogenase DHS-16 to create lathosterone (Rottiers *et al.* 2006; Wollam *et al.* 2012). Lathosterone is a direct Δ^7^-DA precursor and a substrate for the cytochrome P450 family member DAF-9 (Motola *et al.* 2006). The enzyme that catalyzes the conversion of 7-DHC into lathosterol has not been identified.

**Figure 1:**
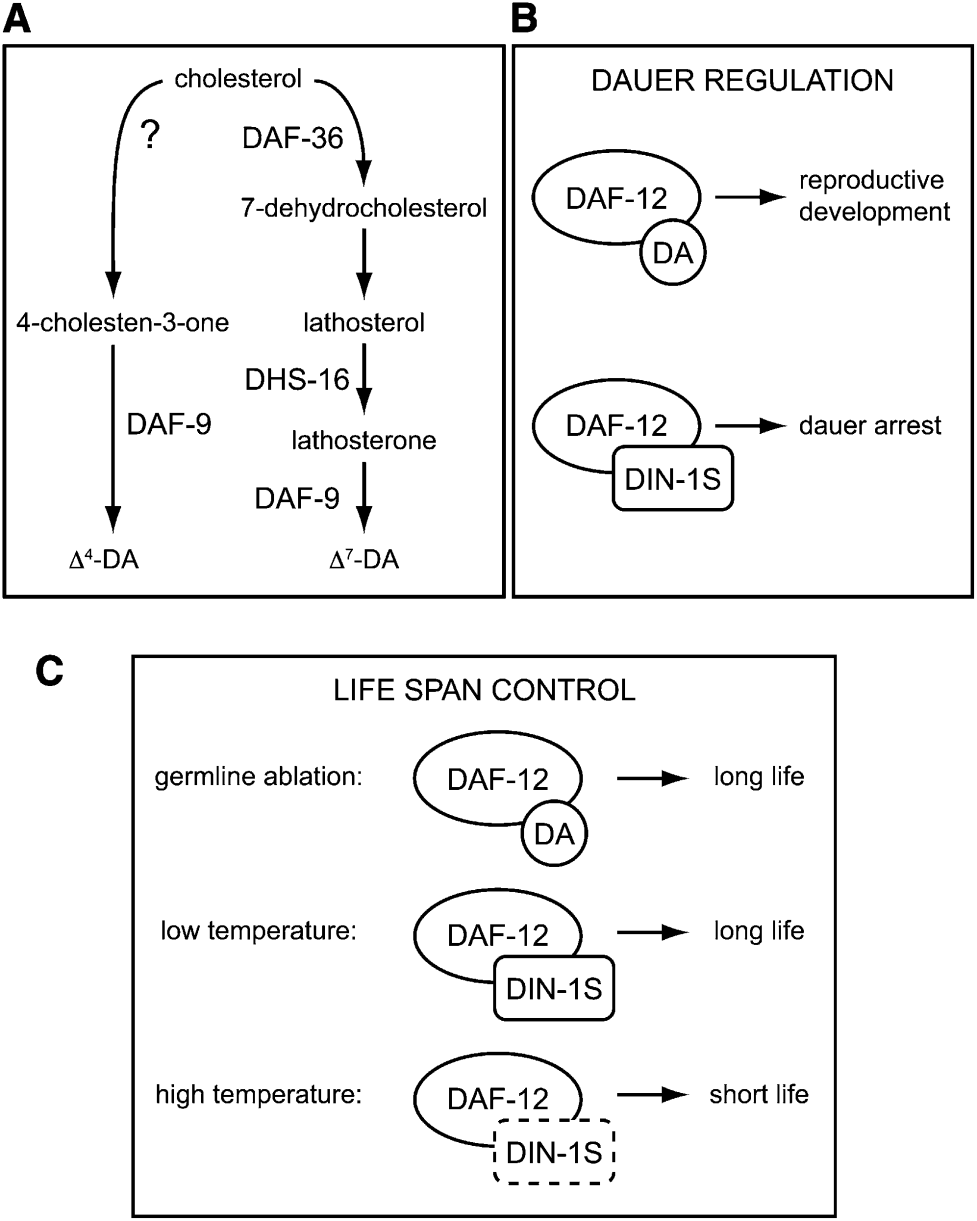
Models of dafachronic acid (DA) biosynthetic pathways and DAF-12 complexes in the control of dauer arrest and life span. (A) Hypothetical model of DA biosynthesis adapted from Wollam *et al.* (2012). (B) Liganded DAF-12 promotes reproductive development, whereas unliganded DAF-12 acts with DIN-1S to promote dauer arrest. (C) Liganded DAF-12 promotes longevity in animals lacking a germline. Unliganded DAF-12 acts with DIN-1S to promote longevity at low temperatures (15°) but shortens life span at higher temperatures (20°–25°). The role of DIN-1S in life span control at higher temperatures is not known.

DAF-9 catalyzes the final common step of DA biosynthesis, converting lathosterone into Δ^7^-DA and 4-cholesten-3-one into Δ^4^-DA (Motola *et al.* 2006). Whereas Δ^7^-DA is detectable in lipid extracts from wild-type *C. elegans*, it is not detectable in extracts from *daf-36* or *daf-9* mutants, indicating that both DAF-36 and DAF-9 are required for Δ^7^-DA synthesis *in vivo* (Motola *et al.* 2006; Wollam *et al.* 2011). Δ^4^-DA has not been unequivocally identified in *C. elegans* extracts.

DAs and DAF-12 have multiple functions during larval development. Under conditions of high population density, food scarcity, and high temperature, wild-type *C. elegans* larvae undergo developmental arrest in an alternative third larval stage known as dauer. Dauer larvae are long-lived and resistant to environmental insults (Hu 2007). *daf-9* mutants, which lack endogenous DAs (Motola *et al.* 2006), arrest as dauer larvae constitutively, even when ambient conditions favor reproductive development (Gerisch *et al.* 2001; Jia *et al.* 2002). This dauer-constitutive phenotype is fully suppressed by exogenous DA (Giroux *et al.* 2008; Motola *et al.* 2006; Sharma *et al.* 2009) as well as by null mutations in *daf-12* (Gerisch *et al.* 2001). *daf-12* ligand binding domain mutants also have a dauer-constitutive phenotype (Antebi *et al.* 1998; Antebi *et al.* 2000). Therefore, unliganded DAF-12 promotes dauer arrest. The dauer-constitutive phenotype of *daf-9* mutants and *daf-12* ligand binding domain mutants is also suppressed by mutations in *din-1S*, which encodes a transcriptional coregulator that binds to DAF-12 (Ludewig *et al.* 2004). Taken together, these results support a model whereby unliganded DAF-12 acts together with DIN-1S to promote dauer arrest; DAs permit reproductive development by binding to DAF-12, thereby preventing its interaction with DIN-1S (Figure 1B) (Fielenbach and Antebi 2008). DAs are also required during larval development for proper gonadal migration (Gerisch *et al.* 2001; Motola *et al.* 2006) and expression of *let-7*-family microRNAs that coordinate the timing of cell divisions (Bethke *et al.* 2009; Hammell *et al.* 2009). In adult males, DAs are required for normal mate searching behavior (Kleemann *et al.* 2008).

The roles of DAs and DAF-12 in the control of adult life span are complex. *daf-9* mutants are long-lived when cultured at 15° (Gerisch *et al.* 2007; Jia *et al.* 2002) but short-lived when cultured at temperatures between 20° and 25° (Gerisch *et al.* 2007; Gerisch *et al.* 2001; Jia *et al.* 2002; Lee and Kenyon 2009). These temperature-dependent phenotypes are suppressed by *daf-12* loss-of-function mutations (Jia *et al.* 2002; Lee and Kenyon 2009) and exogenous DA (Gerisch *et al.* 2007), suggesting that unliganded DAF-12 promotes longevity at low temperatures but shortens life span at higher temperatures (Figure 1C). *din-1S* mutation suppresses the life span extension conferred by *daf-9* mutation at low temperatures (Ludewig *et al.* 2004), indicating that at 15°, unliganded DAF-12 and DIN-1S act together to extend life span (Figure 1C).

DAs and DAF-12 have a profound influence on life span in animals lacking a germline. Ablation of the germline extends adult life span at 20° by ∼60%, and this life span extension requires DAF-9, DAF-36, DAF-12, and the FoxO transcription factor DAF-16 (Gerisch *et al.* 2007; Gerisch *et al.* 2001; Hsin and Kenyon 1999). Exogenous DA restores life span extension in germline-ablated animals harboring *daf-9* or *daf-36* mutations (Gerisch *et al.* 2007), indicating that liganded DAF-12 promotes longevity in this context (Figure 1C).

Similar to germline ablation, loss-of-function mutations in *daf-2*, which encodes the sole *C. elegans* insulin/insulin-like growth factor receptor family member (InsR) (Kimura *et al.* 1997), extend *C. elegans* life span in a DAF-16/FoxO-dependent manner (Kenyon *et al.* 1993). Both DAF-2/InsR and the germline inhibit DAF-16/FoxO activity by promoting its translocation from the nucleus to the cytoplasm (Henderson and Johnson 2001; Lee *et al.* 2001; Lin *et al.* 2001). In mutants that either lack a germline or have reduced DAF-2/InsR signaling, DAF-16/FoxO enters the nucleus, activating a gene regulatory program that promotes longevity (McCormick *et al.* 2012; Murphy *et al.* 2003).

How DAs and DAF-12 influence life span in the context of reduced DAF-2/InsR signaling is poorly understood. The *daf-9* missense allele *rh50* has distinct effects on life span in the context of specific *daf-2* mutant alleles. At 15°, *daf-9*(*rh50*) shortens the life span of both *daf-2*(*e1368*) (harboring a missense mutation in the DAF-2 ligand binding domain) and *daf-2*(*e1370*) (harboring a missense mutation in the tyrosine kinase domain) mutant animals. However, at 22.5°, *daf-9*(*rh50*) shortens *daf-2*(*e1368*) life span but lengthens *daf-2*(*e1370*) life span (Gerisch *et al.* 2001). Accordingly, exogenous Δ^4^-DA prolongs the life span of *daf-2*(*e1368*) animals but does not significantly influence the life span of *daf-2*(*e1370*) animals (Gerisch *et al.* 2007). Furthermore, *daf-12* mutant alleles influence the life span of *daf-2/InsR* mutants in an allele-specific manner. For example, the non-null allele *daf-12*(*m20*) (see Supporting Information, Figure S1) (Antebi *et al.* 2000; Snow and Larsen 2000) suppresses the extended life span phenotype of *daf-2*(*e1368*) harboring a mutation in the ligand binding domain (Patel *et al.* 2008) at all temperatures tested (Gems *et al.* 1998), whereas it enhances *daf-2*(*e1370*) life span extension at high temperatures (Gems *et al.* 1998; Larsen *et al.* 1995). In aggregate, these data underscore the need for further investigation into how steroid hormone signaling and DAF-2/InsR signaling interact in life span control. Specifically, the relative contributions of liganded and unliganded DAF-12 to life span control have not been defined. Prior studies on the interactions of *daf-12* and *daf-2/InsR* mutants in life span control were performed with non-null alleles of *daf-12* (Gems *et al.* 1998; Larsen *et al.* 1995), complicating the interpretation of these experiments.

Here we used null alleles of *daf-36* and *daf-12* to explore the relationship between DA pathways and DAF-2/InsR signaling in life span regulation. Our results are consistent with a model whereby both liganded and unliganded DAF-12 influence life span. Liganded DAF-12 promotes longevity in animals with reduced DAF-2/InsR signaling. Unliganded DAF-12 also extends life span in animals subjected to *daf-2/InsR* RNA interference (RNAi) but shortens life span in *daf-2/InsR* mutants and in animals lacking a germline. These findings establish that distinct DAF-12 activities interact with DAF-2/InsR signaling to control life span.

## Materials and Methods

### *C. elegans* strains

The wild-type N2 Bristol strain was used. Mutant alleles used are described in Table S1. Compound mutants were constructed using standard techniques.

### Dauer arrest assays

Dauer arrest assays were performed at the indicated temperatures in I-36NL model incubators (Percival Scientific, Inc., Perry, IA) as described previously (Hu *et al.* 2006). *P* values were calculated using the Student *t*-test. Statistical analysis of all data is presented in Table S2.

### Life span assays

Life span assays were performed in I-36NL incubators (Percival) at the indicated temperatures. After alkaline hypochlorite treatment and two generations of growth, young adult animals were placed onto nematode growth media (NGM) plates containing 25 μg/ml (100 μM) 5-fluoro-2′-deoxyuridine (FUDR; Sigma) and 10 μg/ml nystatin (Sigma) that had been seeded with 20× concentrated *Escherichia coli* OP50. For life span assays of strains carrying *glp-1*(*e2141*), animals were raised at 25°, and sterile young adult animals were placed onto NGM plates containing nystatin but lacking FUDR as described above. Assays were conducted at 20° unless otherwise noted. Viability was assessed visually or with gentle prodding. Prism software (GraphPad Software, La Jolla, CA) was used for data representation and statistical analysis. *P* values were calculated using the log-rank test. Statistical analysis of all data is presented in Table S2.

### RNAi

Feeding RNAi was performed using variations of standard procedures (Boulton *et al.* 2002). For dauer assays, NGM plates containing 5 mM isopropyl beta-D-1-thiogalactopyranoside (IPTG) and 25 μg/ml carbenicillin were seeded with 500 μl of overnight culture of *E. coli* HT115 harboring either control L4440 vector or *daf-2* RNAi plasmid. Gravid animals cultured on control or *daf-2* RNAi plates were picked to assay plates for 6-hr egg lays. Dauer larvae were scored after progeny had been incubated at 25° for 48–60 hr. For life span assays, NGM plates containing 5 mM IPTG, 25 μg/ml carbenicillin, 25 μg/ml FUDR, and 10 μg/ml nystatin were seeded with 500 μl of 5× concentrated overnight culture of *E. coli* HT115 harboring either control L4440 vector or *daf-2* RNAi plasmid. Young adult animals cultured on standard NGM plates seeded with *E. coli* OP50 were picked to RNAi plates and scored for viability as described above.

## Results

### Modulation of DAF-2/InsR signaling by DAF-12

Two classes of *daf-2* mutants have distinct interactions with the non-null *daf-12*(*m20*) allele (Gems *et al.* 1998). The dauer-constitutive phenotype of Class 1 *daf-2* alleles (*e.g.*, the ligand binding domain mutant *e1368*), which are also long-lived and thermotolerant (Gems *et al.* 1998), is suppressed by *daf-12*(*m20*). In contrast, Class 2 *daf-2* alleles (*e.g.*, the tyrosine kinase domain mutant *e1370*), which have pleiotropic characteristics in addition to the aforementioned Class 1 phenotypes, have a synthetic non-dauer larval arrest phenotype in combination with *daf-12*(*m20*) (Gems *et al.* 1998; Larsen *et al.* 1995; Vowels and Thomas 1992).

Notably, *daf-12*(*m20*) is a nonsense mutation that specifically affects DAF-12A isoforms; it is predicted to truncate DAF-12A upstream of the C-terminal ligand binding domain, potentially resulting in a DAF-12A polypeptide that contains an intact zinc finger in the N-terminal DNA binding domain. The DAF-12B isoform, which contains the ligand binding domain but lacks the DNA binding domain, is not affected by *m20* (Figure S1) (Antebi *et al.* 2000; Snow and Larsen 2000). The influence of a *daf-12* null allele on the dauer-constitutive phenotype of *daf-2* mutants has not been explored.

To clarify the epistatic relationship between *daf-2* and *daf-12*, we constructed *daf-2;daf-12* double mutants using the *daf-12*(*rh61rh411*) null allele (Figure S1 and Table S1) hereafter referred to as “*daf-12* null” (Antebi *et al.* 2000; Snow and Larsen 2000) and performed dauer arrest assays at 25°. As expected, both the Class 1 *daf-2*(*e1368*) ligand binding domain mutant and the Class 2 *daf-2*(*e1370*) tyrosine kinase domain mutant had strong dauer-constitutive phenotypes (Figure S2). The dauer-constitutive phenotype of *daf-2*(*e1368*) was completely suppressed by *daf-12*(*null*), consistent with the effect of *daf-12*(*m20*) on other Class 1 *daf-2* alleles (Gems *et al.* 1998). At 15°, *daf-2*(*e1370*)*;daf-12*(*null*) double mutants developed reproductively into adults (data not shown). At 25°, they arrested as larvae that were longer and wider than dauer larvae and that lacked both dauer alae and pharyngeal remodeling (Figure S2 and data not shown). This phenotype is comparable to that previously described for *daf-2*(*e1370*)*;daf-12*(*m20*) double mutants (Gems *et al.* 1998; Larsen *et al.* 1995; Vowels and Thomas 1992) and suggests that the DAF-12A isoforms that are affected by the *m20* mutation are the isoforms that prevent non-dauer larval arrest in *daf-2*(*e1370*) mutants at 25°.

**Figure 2:**
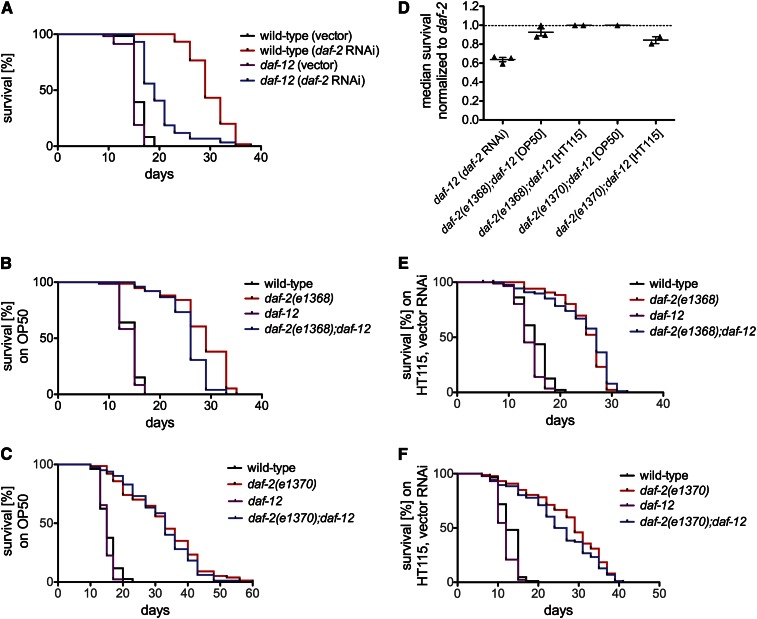
Modulation of life span by *daf-12*(*null*) mutation in animals with reduced DAF-2/InsR activity. (A) In the context of DAF-2/InsR activity reduction by *daf-2* RNAi, the *daf-12*(*null*) mutation shortened median life span [*daf-12*(*null*) *vs.* that of the wild-type, *P* < 0.0001]. (B) In the context of the Class 1 *daf-2*(*e1368*) allele, *daf-12*(*null*) shortened the median life span [*daf-2*(*e1368*)*;daf-12*(*null*) *vs. daf-2*(*e1368*), *P* < 0.0001]. (C) The *daf-12*(*null*) mutation did not shorten the median life span of animals with DAF-2/InsR activity reduction via the Class 2 *daf-2*(*e1370*) allele [*daf-2*(*e1370*)*;daf-12*(*null*) *vs. daf-2*(*e1370*), *P* = 0.4275]. (D) Scatter plot of median survival of *daf-12*(*null*) animals normalized to that of *daf-12* wild-type animals in the three contexts of reduced DAF-2/InsR activity, separated by assay food source. Error bars indicate SEM. (E and F) Food source control experiments described in (B) and (C), respectively. *E. coli* HT115 expressing vector control RNAi was used as the assay food source as opposed to *E. coli* OP50. For each experiment, more than 60 animals were scored per genotype, and at least two experimental replicates were performed. See Table S2 for all raw data and statistics.

*daf-12*(*m20*) also has disparate effects on the longevity of Class 1 and Class 2 *daf-2* mutants (Gems *et al.* 1998; Larsen *et al.* 1995). To gain insight into how DAF-12 influences life span in animals with reduced DAF-2/InsR signaling, we measured life spans of *daf-12*(*null*) animals in three contexts of reduced DAF-2/InsR activity. First, we performed *daf-2* RNAi in wild-type and *daf-12*(*null*) animals. *daf-2* RNAi does not induce dauer arrest in wild-type animals at 25° but enhances dauer arrest at 27° (Dillin *et al.* 2002), suggesting that the extent to which RNAi reduces DAF-2 activity is less than that caused by Class 1 and Class 2 *daf-2* mutant alleles, which all have strong dauer-constitutive phenotypes at 25° (Gems *et al.* 1998). As previously observed (Dillin *et al.* 2002), *daf-2* RNAi extended life span to a degree comparable to *daf-2* mutation (Figure 2, A–C). Life span extension induced by *daf-2* RNAi was significantly attenuated in *daf-12*(*null*) animals (Figure 2, A and D; Table S2); *daf-12*(*null*) animals subjected to *daf-2* RNAi exhibited a 34.5% decrease in median survival compared to wild-type animals on *daf-2* RNAi (*P* < 0.0001, log-rank test), suggesting that DAF-12 is required for life span extension in animals with reduced DAF-2/InsR activity. At 25°, *daf-12*(*null*) mutants live approximately as long as wild-type animals do (Figure 2A, *P* = 0.0018; Figure 2B, *P* = 0.1787; Figure 2C, *P* = 0.0678; Table S2), indicating that the effect of *daf-12*(*null*) on the life span of animals subjected to *daf-2* RNAi is unlikely to be due to general frailty. Furthermore, RNAi of three unrelated genes in wild-type and *daf-12*(*null*) animals revealed that *daf-12*(*null*) animals do not have an Rde (RNAi-defective) phenotype (Figure S3). This indicates that the relative reduction in life span extension caused by *daf-2* RNAi in *daf-12*(*null*) animals is unlikely to be due to reduced inactivation of *daf-2*.

We also assayed the life spans of *daf-2;daf-12*(*null*) double mutants. Interestingly, although *daf-2*(*e1368*)*;daf-12*(*null*) animals had a shorter life span than Class 1 *daf-2*(*e1368*) single mutants, the effect of *daf-12*(*null*) on life span extension in the context of the Class 1 *daf-2*(*e1368*) mutation was significantly smaller than its effect in the context of *daf-2* RNAi [Figure 2, B and D, and Table S2: *daf-2*(*e1368*)*;daf-12*(*null*) exhibited a 10.3% decrease in median survival compared to *daf-2*(*1368*) alone (*P* < 0.0001)]. Furthermore, *daf-2*(*e1370*)*;daf-12*(*null*) animals lived as long as Class 2 *daf-2*(*e1370*) single mutants [Figure 2, C and D, and Table S2: 0% change in median survival of *daf-2*(*e1370*)*;daf-12*(*null*) compared to *daf-2*(*e1370*), *P* = 0.4275]. Taken together, these results suggest that DAF-12 is required for life span extension in the context of RNAi knock-down of *daf-2*, but is largely dispensable for longevity in the context of Class I *daf-2*(*e1368*) and Class II *daf-2*(*e1370*) mutants (see comparison of replicate experiments in Figure 2D).

A possible explanation for the differences in the influence of DAF-12 on life span between the contexts of *daf-2* RNAi and mutational reduction of DAF-2/InsR activity is the distinct food sources employed in each experimental condition. RNAi by feeding, as used to reduce *daf-2/InsR* activity, involves the use of an *E. coli* strain, HT115, which is distinct from the standard lab food source, *E. coli* OP50. It has been shown that using HT115 in place of OP50 as a food source is sufficient to impact *C. elegans* longevity (Maier *et al.* 2010). To test whether *E. coli* strain differences influence the effect of *daf-12*(*null*) on life span in the context of reduced DAF-2/InsR activity, we performed life span assays with *daf-2*(*e1368*) and *daf-2*(*e1370*) mutant animals grown on *E. coli* HT115 (expressing empty vector control RNAi) as the food source. Under these conditions, *daf-2*(*e1368*)*;daf-12* double mutants were not shorter lived than *daf-2*(*e1368*) single mutants [Figures 2, D and E, and Table S2: *daf-2*(*e1368*)*;daf-12* animals exhibited a 0% change in median life span compared to *daf-2*(*e1368*), *P* = 0.1989]. *daf-2*(*e1370*)*;daf-12* double mutants grown on *E. coli* HT115 were shorter lived than *daf-2*(*e1370*) single mutants, but the difference in median life span was not statistically significant [Figures 2, D and F, and Table S2: 12.1% decrease in median life span compared to *daf-2*(*e1370*), *P* = 0.1439]. These results suggest that the food source does not account for the differential effects of *daf-12*(*null*) on longevity in the three contexts of reduced *daf-2/InsR* activity that we examined.

### Modulation of DAF-2/InsR signaling by DA biosynthetic components

**Figure 3:**
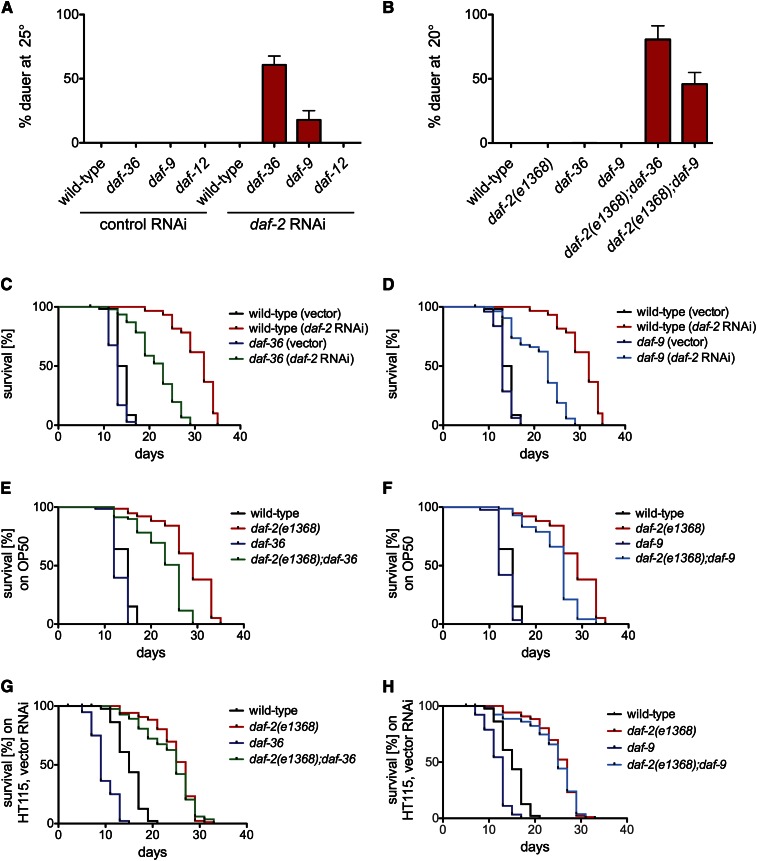
Mutations that reduce DA biosynthesis promote dauer arrest and shorten life span in animals with reduced DAF-2/InsR signaling. (A and B) *daf-36*(*null*) and *daf-9*(*k182*) mutations enhance dauer arrest of animals subjected to *daf-2* RNAi (A) [wild-type on *daf-2* RNAi *vs. daf-36*(*null*) on *daf-2* RNAi, *P* = 0.0009; wild-type on *daf-2* RNAi *vs. daf-9*(*k182*) on *daf-2* RNAi, *P* = 0.0689], or harboring the Class I *daf-2*(*e1368*) allele]; (B) [*daf-2*(*e1368*) *vs. daf-2*(*e1368*)*;daf-36*(*null*), *P* = 0.0017; *daf-2*(*e1368*) *vs. daf-2*(*e1368*)*;daf-9*(*k182*), *P* = 0.0072]. Data represent the averages of three replicate experiments with a minimum of 400 animals scored per genotype. Error bars indicate SEM. (C and D) *daf-36*(*null*) or *daf-9*(*k182*) mutations reduce life span of animals subjected to *daf-2* RNAi [*P* < 0.0001] or (E and F) harboring the Class I *daf-2*(*e1368*) allele [*P* < 0.0001]. (G and H) Food source control experiments for (E) and (F), respectively. *E. coli* HT115 expressing vector control RNAi was used as the assay food source as opposed to *E. coli* OP50. For each life span experiment, more than 60 animals were assayed per genotype. All raw data and statistics, including data from experimental replicates, are presented in Table S2.

Since DAF-12 is regulated by DA ligands (Motola *et al.* 2006), we explored the influence of mutations in DA biosynthetic pathway components on dauer arrest and life span in animals with reduced DAF-2/InsR activity. Mutations in two genes encoding components of DA biosynthetic pathways, *daf-36* and *daf-9*, cause a dauer-constitutive phenotype (Gerisch *et al.* 2001; Jia *et al.* 2002; Rottiers *et al.* 2006). The null allele *daf-36*(*k114*) (Rottiers *et al.* 2006) (heretofore referred to as “*daf-36*(*null*)”) and the partial loss-of-function allele *daf-9*(*k182*) enhanced the dauer-constitutive phenotype induced by *daf-2* RNAi at 25° (Figure 3A). They also enhanced the dauer-constitutive phenotype of the Class 1 *daf-2*(*e1368*) ligand binding domain mutant at 20° (Figure 3B) and 15° (Figure S4).

To elucidate interactions between DA biosynthetic pathways and DAF-2/InsR signaling in life span control, we performed life span assays in *daf-36*(*null*) and *daf-9*(*k182*) mutants in two contexts of reduced DAF-2/InsR activity: *daf-2* RNAi and *daf-2*(*e1368*)*. daf-36*(*null*) and *daf-9*(*k182*) mutations both reduced life span extension induced by *daf-2* RNAi [Figure 3C: *daf-36*(*null*) exhibited a 25.8% decrease in median life span compared to wild-type animals on *daf-2* RNAi, *P* < 0.0001; Figure 3D: *daf-9*(*k182*) exhibited a 28.1% decrease in median life span compared to wild-type, *P* < 0.0001; Table S2]. Because neither Δ^4^- nor Δ^7^-DA is detectable in *daf-36* null mutants (Wollam *et al.* 2011), these results suggest that DAs are required for maximal life span extension in animals subjected to *daf-2* RNAi. Similar to the case for DAF-12 (Figure 2), the requirement for DA biosynthesis in life span extension induced by reduced DAF-2/InsR activity is context-dependent, as the magnitude of life span reduction caused by *daf-36* and *daf-9* mutations was smaller in animals harboring the Class 1 *daf-2*(*e1368*) allele than in animals subjected to *daf-2* RNAi [Figure 3E: *daf-2*(*e1368*)*;daf-36*(*null*) median life span was 10.3% less than that of *daf-2*(*e1368*), *P* < 0.0001; Figure 3F: *daf-2*(*e1368*)*;daf-9*(*k182*) median life span was 10.3% less than that of *daf-2*(*e1368*), *P* < 0.0001; compare to Figures 3, C and D; Table S2; results summarized in Table 1]. The difference in response on *daf-2* RNAi compared to *daf-2/InsR* genetic mutation was not due to differences in the *E. coli* strain used as a food source, as *daf-2*(*e1368*) mutant animals had similar life spans when assayed on *E. coli* HT115 (with vector control RNAi) and *E. coli* OP50 [Figure 3G: on HT115, median life span of *daf-2*(*e1368*)*;daf-36*(*null*) animals was 7.4% shorter than *daf-2*(*e1368*), *P* = 0.4328; Figure 3H: on HT115, median life span of *daf-2*(*e1368*)*;daf-9*(*k182*) animals was 7.4% shorter than *daf-2*(*e1368*), *P* = 0.2991; Table S2]. Collectively, these data suggest that both DAs and DAF-12 contribute to life span extension in animals with reduced DAF-2/InsR activity.

**Table 1:** Summary of effects of *daf-12* and *daf-36* null mutations on life span in three contexts of DAF-16/FoxO activation

Genetic Context	Percentage of Life Span Shortened by *daf-12*(*null*) [*P* value]	Percentage of Life Span Shortened by *daf-36*(*null*) [*P* value]	Effect of *daf-12*(*null*) Mutation on Life Span in *daf-36*(*null*) [*P* value]	Effect of Liganded DAF-12 on Life Span	Effect of Unliganded DAF-12 on Life Span
*daf-2* RNAi	34.5 (<0.0001)	10.0 (<0.0001)	25.9 (<0.0001) ↓	↑	↑
	(Figure 2A)	(Figure 4A)	(Figure 4A)		
*daf-2*(*e1368*)	10.3 (<0.0001)	29.4 (<0.0001)	29.2 (<0.0001) ↑	↑	↓
	(Figure 2B)	(Figure 4B)	(Figure 4B)		
*glp-1*(*e2141*)	60.7 (<0.0001)	60.7 (<0.0001)	27.3 (<0.0001) ↑	↑	↓
	(Figure 4D)	(Figure 4D)	(Figure 4D)		

*daf-2* RNAi, *daf-2*(*e1368*) mutation and germline ablation [*glp-1*(*e2141*) animals, raised at the restrictive temperature] were used to induce DAF-16/FoxO-dependent life span extension. Percentage of changes in median life span *vs.* the comparator (*P* values) are shown for each indicated experiment. See Table S2 for replicate experiments. Arrows indicate the direction of effect on life span (↓, decrease; ↑, increase). The two right columns show the qualitative effects of liganded and unliganded DAF-12 on life span.

### Role of unliganded DAF-12 in life span control by DAF-2/InsR signaling

To elucidate the relative contributions of liganded and unliganded DAF-12 to life span control in animals with reduced DAF-2/InsR signaling, we determined the influence of *daf-12*(*null*) mutation on the life spans of *daf-36*(*null*) animals with reduced DAF-2/InsR activity. Since *daf-36*(*null*) animals do not make Δ^4^- or Δ^7^-DA (Wollam *et al.* 2011), DAF-12 activity in the context of *daf-36*(*null*) is largely attributable to unliganded DAF-12.

**Figure 4:**
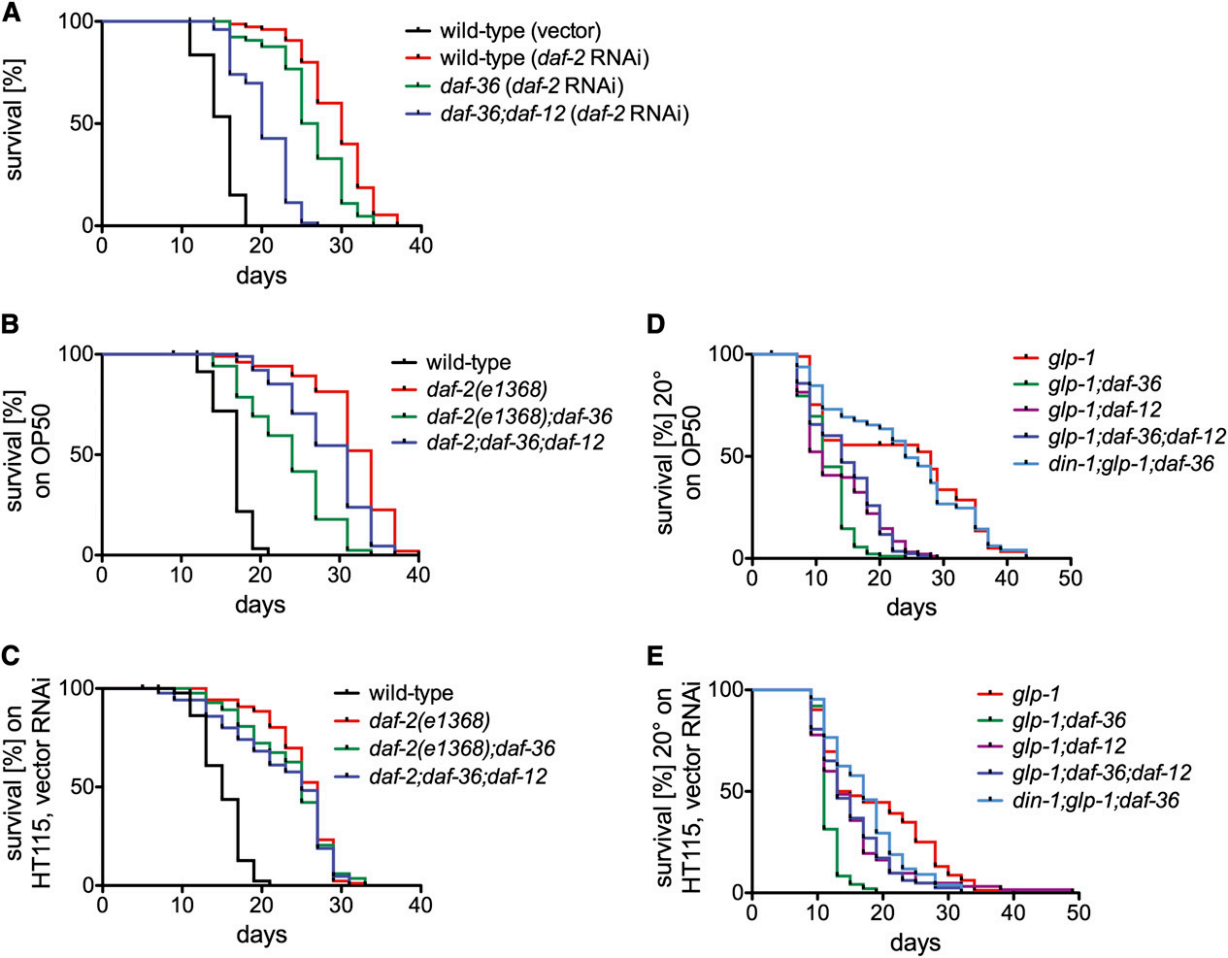
Unliganded DAF-12 influences life span in a context-dependent manner. (A) *daf-12*(*null*) mutation further decreased median life span of *daf-36*(*null*) animals on *daf-2* RNAi [*daf-36*(*null*)*;daf-12*(*null*) *vs. daf-36*(*null*), *P* < 0.0001]. (B and D) In contrast, *daf-12*(*null*) mutation increased median life span of *daf-36*(*null*) in the context of *daf-2*(*e1368*), (B) and germline ablation (*glp-1* mutation), (D) [*daf-2*(*e1368*)*;daf-36*(*null*)*;daf-12*(*null*) *vs. daf-2*(*e1368*)*;daf-36*(*null*), *P* < 0.0001; *glp-1;daf-36*(*null*)*;daf-12*(*null*) *vs. glp-1;daf-36*(*null*), *P* < 0.0001]. (D) *din-1S*(*null*) abrogated life span shortening induced by *daf-36*(*null*) in the context of germline ablation [*din-1S*(*null*)*;glp-1;daf-36*(*null*) *vs. glp-1;daf-36*(*null*), *P* < 0.0001]. (C and E) Food source control experiments for (B) and (D), respectively. *E. coli* HT115 expressing vector control RNAi was used as the assay food source as opposed to *E. coli* OP50. For each experiment, more than 60 animals were assayed per genotype, and at least two experimental replicates were performed. Raw data and statistics are presented in Table S2.

In animals subjected to *daf-2* RNAi, *daf-36*(*null*) mutation reduced life span (Figures 3C and 4A), as did the hypomorphic *daf-9*(*k182*) mutation (Figure 3D). *daf-36*(*null*)*;daf-12*(*null*) animals subjected to *daf-2* RNAi had even shorter life spans than *daf-36*(*null*) single mutants subjected to *daf-2* RNAi [Figure 4A and Table S2: *daf-36*(*null*)*;daf-12*(*null*) had a 25.9% decrease in median life span compared to *daf-36*(*null*) on *daf-2* RNAi, *P* < 0.0001]. From this finding, we infer that unliganded DAF-12 promotes longevity in the context of *daf-2* RNAi.

In the *daf-2*(*e1368*) background, *daf-36*(*null*) and *daf-9*(*k182*) mutations also reduced life span (Figures 3, E and F, and 4B). However, in contrast to our findings with *daf-2* RNAi, *daf-12*(*null*) mutation did not further shorten the life spans of *daf-2*(*e1368*)*;daf-36*(*null*) double mutant animals. Whereas *daf-12*(*null*) mutation shortened the life span of *daf-36*(*null*) animals subjected to *daf-2* RNAi (Figure 4A, 25.9% decrease in median life span, *P* < 0.0001), it extended the life span of *daf-2*(*e1368*)*;daf-36*(*null*) animals fed *E. coli* OP50 [Figure 4B and Table S2: 29.2% increase in median life span of *daf-2*(*e1368*)*;daf-36*(*null*)*;daf-12*(*null*) compared to *daf-2*(*e1368*)*;daf-36*(*null*), *P* < 0.0001]. The food source does not account for this difference; in contrast to the context of *daf-2* RNAi, *daf-12*(*null*) mutation did not shorten the life spans of *daf-2*(*e1368*)*;daf-36*(*null*) mutant animals when animals were grown on *E. coli* HT115 [Figure 4C and Table S2; 0% change in median life span comparing *daf-2*(*e1368*)*;daf-36*(*null*);daf-12(null) to *daf-2*(*e1368*)*;daf-36*(*null*), *P* = 0.6097]. Because *daf-12* null mutation in the context of *daf-2*(*e1368*) mutation and the absence of DA is either beneficial or neutral to life span (Figure 4, B and C), we conclude that unliganded DAF-12 shortens life span in *daf-2*(*e1368*) mutant animals. We were unsuccessful in our efforts to construct *daf-2*(*e1370*)*;daf-36*(*null*) double mutants; this precluded an assessment of the influence of *daf-36*(*null*) mutation on life span in the *daf-2*(*e1370*) mutant background.

In aggregate, our results (summarized in Table 1) support roles for both liganded and unliganded DAF-12 in life span control in animals with reduced DAF-2/InsR signaling. Liganded DAF-12 promotes longevity in all contexts tested, whereas unliganded DAF-12 modulates life span in a context-dependent manner; in the context of reduced DAF-2/InsR signaling via *daf-2* RNAi, unliganded DAF-12 promotes longevity. In contrast, in the context of DAF-2/InsR signaling reduction via *daf-2*(*e1368*) mutation, unliganded DAF-12 is detrimental to life span.

### Role of unliganded DAF-12 in life span control by the germline

Although both DAF-2/InsR and the germline control life span by regulating DAF-16/FoxO activity, they do so through distinct molecular pathways (Berman and Kenyon 2006; Ghazi *et al.* 2009; Hsin and Kenyon 1999). DA biosynthetic enzymes and DAF-12 are required for life span extension induced by germline ablation (Gerisch *et al.* 2007; Gerisch *et al.* 2001; Hsin and Kenyon 1999; Yamawaki *et al.* 2010), suggesting that liganded DAF-12 is important in promoting longevity in animals lacking a germline. In light of our finding that unliganded DAF-12 can shorten life span in *daf-2/InsR* mutants (Figure 4B), we sought to determine whether unliganded DAF-12 also shortens life span in animals lacking a germline.

We confirmed previously established requirements for *daf-36* and *daf-12* in life span extension induced by germline ablation (Figure 4C) (Gerisch *et al.* 2007; Hsin and Kenyon 1999; Yamawaki *et al.* 2010). Notably, in three of five replicate experiments, we found that *glp-1;daf-12*(*null*) animals lived longer than *glp-1;daf-36*(*null*) animals (Figure 4D and Table S2). Because *daf-36*(*null*) animals do not make Δ^4^- or Δ^7^-DA (Wollam *et al.* 2011), this result suggested the possibility that unliganded DAF-12 shortens life span in animals lacking a germline. To determine whether this was the case, we examined the influence of *daf-12*(*null*) mutation on life span in germline-ablated *daf-36*(*null*) animals. *glp-1;daf-36*(*null*)*;daf-12*(*null*) triple-mutation animals lived significantly longer than *glp-1;daf-36*(*null*) double-mutation animals [Figure 4D and Table S2: 27.3% increase in median life span of *glp-1;daf-36*(*null*)*;daf-12*(*null*) compared to *glp-1;daf-36*(*null*), *P* < 0.0001], indicating that unliganded DAF-12 also shortens life span in germline-ablated animals. This result was recapitulated by control experiments performed on *E. coli* HT115 as the food source [Figure 4E and Table S2: 18.2% increase in median life span of *glp-1;daf-36*(*null*)*;daf-12*(*null*) compared to *glp-1;daf-36*(*null*), *P* < 0.0001]. Thus, DAF-12 has at least two distinct activities that control life span in germline-ablated animals: liganded DAF-12 promotes longevity, whereas unliganded DAF-12 shortens life span. These findings are summarized in Table 1.

### Role of the transcriptional coregulator DIN-1S in life span control by the germline

In animals lacking DAs, DIN-1S, the short isoform of the transcriptional coregulator DIN-1, acts in a complex with unliganded DAF-12 to promote dauer arrest (Figure 1B) (Ludewig *et al.* 2004). Since unliganded DAF-12 shortens life span in germline-ablated animals (Figure 4D), we examined the role of DIN-1S in life span control in animals lacking a germline by determining the impact of the *din-1S* null mutation *dh127* (Ludewig *et al.* 2004) (hereafter referred to as “*din-1S*(*null*)”) on the life spans of *glp-1;daf-36*(*null*) double-mutation animals. *din-1S*(*null*) animals have life spans comparable to wild-type animals at 15° (Ludewig *et al.* 2004). Surprisingly, *din-1S*(*null*) completely suppressed the life span shortening effect of *daf-36*(*null*) on germline-ablated animals fed *E. coli* OP50 [Figure 4D and Table S2: *P* = 0.8829 for the comparison of *din-1S*(*null*)*;glp-1;daf-36*(*null*) to *glp-1* single mutant; *din-1S*(*null*)*;glp-1;daf-36*(*null*) median life span was between 35.3% and 118.2% longer than that of *glp-1;daf-36*(*null*) in four replicate experiments, *P* < 0.0001 for each experiment]. This result was replicated with *E. coli* HT115 as the food source [Figure 4E and Table S2: *P* = 0.0389 for the comparison of *din-1S*(*null*)*;glp-1;daf-36*(*null*) to *glp-1* single mutant; *din-1S*(*null*)*;glp-1;daf-36*(*null*) median life span was 54.4% longer than that of *glp-1;daf-36*(*null*), *P* < 0.0001]. Thus, in *daf-36*(*null*) animals lacking a germline, DIN-1S plays a major role in shortening life span.

## Discussion

Although the interface between *C. elegans* hormone signaling and the DAF-2/InsR pathway has been explored previously (Gems *et al.* 1998; Larsen *et al.* 1995), how these pathways interact to influence longevity remains obscure. Our work provides novel insights into the genetic interactions of liganded and unliganded DAF-12 with DAF-2/InsR signaling in life span control.

### Liganded DAF-12 promotes longevity in animals with reduced DAF-2/InsR activity

Ambiguity about the role of DAF-12 in determining longevity is due at least in part to the use of the non-null *daf-12*(*m20*) allele in previous investigations (Gems *et al.* 1998; Larsen *et al.* 1995). We now show that the *daf-12*(*rh61rh411*) null allele and the non-null *daf-12*(*m20*) allele have distinct effects on the life spans of animals with reduced DAF-2/InsR signaling (Figure 2) (Gems *et al.* 1998; Larsen *et al.* 1995; McCulloch and Gems 2007). Our results indicate that at high temperatures, DAF-12 promotes longevity in animals with reduced DAF-2/InsR signaling (Figure 2). The magnitude of this life-span-extending effect of DAF-12 is greater in animals subjected to *daf-2* RNAi than in animals harboring *daf-2* mutation, indicating that the specific context of reduced DAF-2/InsR activity influences the role of DAF-12 in life span control (Table 1). The disparity between our results and those obtained with the non-null *daf-12*(*m20*) allele (Gems *et al.* 1998; Larsen *et al.* 1995) suggests that the longevity-promoting effect of *daf-12*(*m20*) and other non-null *daf-12* mutations (that specifically affect DAF-12A isoforms) on the life span of *daf-2*(*e1370*) and other Class 2 *daf-2* mutants (Antebi *et al.* 2000; Gems *et al.* 1998; Larsen *et al.* 1995; McCulloch and Gems 2007) may be attributable to a life-span-extending activity of either the DAF-12B isoform, which contains a ligand binding domain but no DNA binding domain, or truncated DAF-12A polypeptides containing most of the DNA binding domain but lacking the ligand binding domain (Antebi *et al.* 2000; Snow and Larsen 2000). These DAF-12 polypeptides do not play a significant role in dauer regulation by DAF-2/InsR, as *daf-12*(*null*) and *daf-12*(*m20*) have similar effects on the dauer-constitutive phenotypes of *daf-2* mutants (Figure S1).

**Figure 5:**
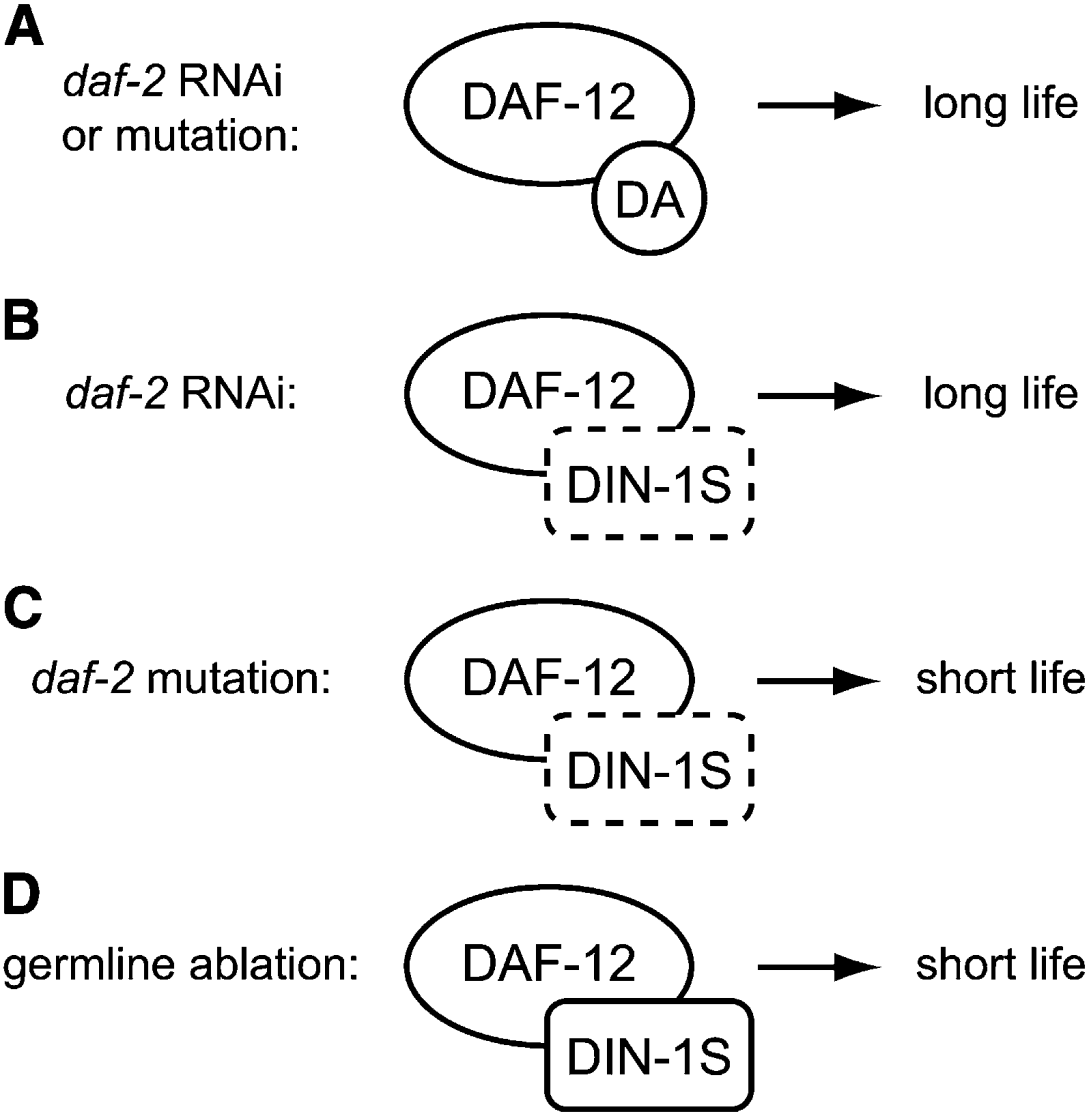
New functions of DAF-12 complexes in life span control. (A) Liganded DAF-12 promotes longevity in animals with reduced DAF-2/InsR activity. (B and C) Unliganded DAF-12 promotes longevity in animals subjected to *daf-2* RNAi (B) but shortens life span in *daf-2* mutant animals (C). (D) Unliganded DAF-12 acts together with DIN-1S to shorten life span in animals lacking a germline.

The observation that mutations in either *daf-12* (Figure 2) or genes encoding DA biosynthetic components (Figure 3) reduce life span in animals with reduced DAF-2/InsR signaling is consistent with a model whereby liganded DAF-12 promotes longevity when DAF-2/InsR signaling is reduced (Figure 5A). Similar results indicate that liganded DAF-12 also promotes longevity in germline-ablated animals (Figure 4D) (Gerisch *et al.* 2007; Gerisch *et al.* 2001; Hsin and Kenyon 1999; Yamawaki *et al.* 2010). The magnitude of the effect of reducing the activity of DA biosynthetic components or DAF-12 on life span is greater in animals lacking a germline than in animals with reduced DAF-2/InsR activity (Figures 2–4) (Gerisch *et al.* 2007; Gerisch *et al.* 2001; Hsin and Kenyon 1999; Yamawaki *et al.* 2010). The molecular basis for this observation is not known.

### Unliganded DAF-12 has context-dependent influences on life span in animals with reduced DAF-2/InsR activity

Unliganded DAF-12 promotes longevity in animals cultured at low temperatures (Gerisch *et al.* 2001; Jia *et al.* 2002) but shortens life span in animals that are cultured at high temperatures (Lee and Kenyon 2009). Here we show that in the context of reduced DAF-2/InsR signaling, unliganded DAF-12 can either extend or shorten life span. In *daf-36*(*null*) animals, which lack both Δ^4^- and Δ^7^-DA (Wollam *et al.* 2011), DAF-12 extends life span in the context of *daf-2* RNAi (Figure 4A) but shortens life span in the contexts of the Class 1 *daf-2*(*e1368*) allele (Figure 4B) and germline ablation (Figure 4D). Since DAF-16/FoxO is a major target of both DAF-2/InsR signaling and germline signaling in life span control (Hsin and Kenyon 1999; Kenyon *et al.* 1993), it is likely that the impact of unliganded DAF-12 on longevity is strongly influenced by relative levels of DAF-16/FoxO activity. This notion is supported by a recent report demonstrating that DAF-12 and DAF-16/FoxO mutually influence target gene expression in animals lacking a germline (McCormick *et al.* 2011).

### Transcriptional coregulator DIN-1S shortens life span in animals lacking a germline

DIN-1S acts together with unliganded DAF-12 at 15° to promote longevity (Ludewig *et al.* 2004). Here we show for the first time that the DAF-12 coregulator DIN-1S plays a major role in life span control in germline-ablated animals. Both *daf-12*(*null*) and *din-1S*(*null*) suppressed the life-span-shortening effect of *daf-36*(*null*) on animals lacking a germline (Figure 4, D and E). This is consistent with a model whereby unliganded DAF-12 and DIN-1S act together to shorten life span. The role of DIN-1S in life span control by unliganded DAF-12 in the context of reduced DAF-2/InsR signaling is not known.

### Context-dependent life span control by DAF-12 complexes

Our results define new functions for DAF-12 complexes in life span control and underscore the context-dependence of these activities (Figure 5). As summarized in Table 1, liganded DAF-12 promotes longevity both in animals with reduced DAF-2/InsR activity (Figures 2, A–C, E and F, and 3, C–H) as well as in animals lacking a germline (Figure 4, D and E) (Gerisch *et al.* 2007; Yamawaki *et al.* 2010). Unliganded DAF-12 also promotes longevity in animals subjected to *daf-2* RNAi (Figure 4A) but shortens life span in animals harboring *daf-2* mutations or lacking a germline (Figure 4, B–E). The basis for the context-dependent influence of unliganded DAF-12 on life span may involve context-specific proteins and/or undiscovered DAF-12 ligands present in *daf-36*(*null*) animals that influence the transcriptional regulatory activity of DAF-12 complexes.

## Supplementary Material

Supporting Information
