# Supplementary material for: Influence of Steroid Hormone Signaling on Life Span Control by Caenorhabditis elegans Insulin-Like Signaling
Source: G3 (Bethesda). 2013 May 1;3(5):841–50. doi: 10.1534/g3.112.005116 (PMC3656731; doi:10.1534/g3.112.005116)
Supplement: Supporting Information [file supp_3_5_841__index.html]

Influence of Steroid Hormone Signaling on Life Span Control by Caenorhabditis elegans Insulin-Like Signaling — Supporting Information 

# Influence of Steroid Hormone Signaling on Life Span Control by *Caenorhabditis elegans* Insulin-Like Signaling

## Supporting Information for Dumas *et al.*, 2013

**Files in this Data Supplement:**

- Supporting Information - Figures S1-S4 and Tables S1 and S2 (PDF, 1.3 MB)
- Figure S1 - *daf-12* gene structure, transcripts, and relevant mutations (PDF, 872 KB)
- Figure S2 - Larval arrest phenotypes of *daf-2;daf-12(null)* double mutants at 25 degree (PDF, 172 KB)
- Figure S3 - *daf-12(null)* mutation does not cause an RNAi-defective phenotype (PDF, 241 KB)
- Figure S4 - Enhancement of the 15 degree dauer-constitutive phenotype *daf-2(e1368)* by mutations in genes encoding DA biosynthetic pathway components (PDF, 115 KB)
- Table S1 - Mutant alleles used in this study (PDF, 63 KB)
- Table S2 - Statistical analysis of all data (.xlsx, 76 KB)
