# Supplementary material for: Influence of Steroid Hormone Signaling on Life Span Control by Caenorhabditis elegans Insulin-Like Signaling
Source: G3 (Bethesda). 2013 May 1;3(5):841–50. doi: 10.1534/g3.112.005116 (PMC3656731; doi:10.1534/g3.112.005116)
Supplement: Supporting Information [file supp_g3.112.005116_005116SI.pdf]

G3·Genes | Genomes | Genetics

The influence of steroid hormone signaling on life span control by *Caenorhabditis elegans* insulin-like signaling

Kathleen J. Dumas[*], Chunfang Guo, Hung-Jen Shih, and Patrick J. Hu[§]

Life Sciences Institute, University of Michigan

[*]Cellular and Molecular Biology Graduate Program

[§]Departments of Internal Medicine and Cell and Developmental Biology

University of Michigan Medical School

Ann Arbor, MI 48109, USA

DOI: 10.1534/g3.112.005116

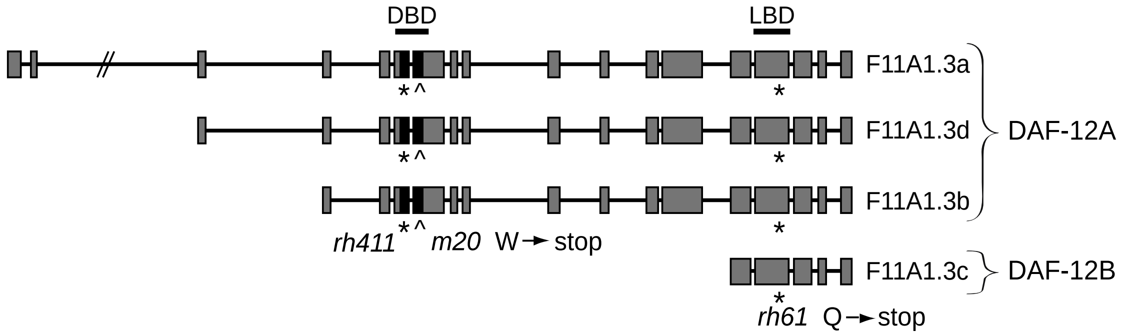

Figure S1 ***daf-12*** **gene structure, transcripts, and relevant mutations.** The structure of *daf-12* transcripts is adapted from WormBase (WBGene00000908). Transcripts corresponding to DAF-12A and DAF-12B isoforms as first described in Antebi *et al.* 2000 and Snow and Larsen 2000 are shown. The location of molecular lesions is taken from Antebi *et al.* 2000 and Snow and Larsen 2000. The *m20* mutation is indicated by a caret, and the *rh411* mutation is indicated by an asterisk in the DNA binding domain. *rh411* is a small deletion/duplication after the first Zn finger in the DNA binding domain that results in an in-frame stop (Antebi *et al.* 2000). Abbreviations: DBD, DNA binding domain; LBD, ligand binding domain.

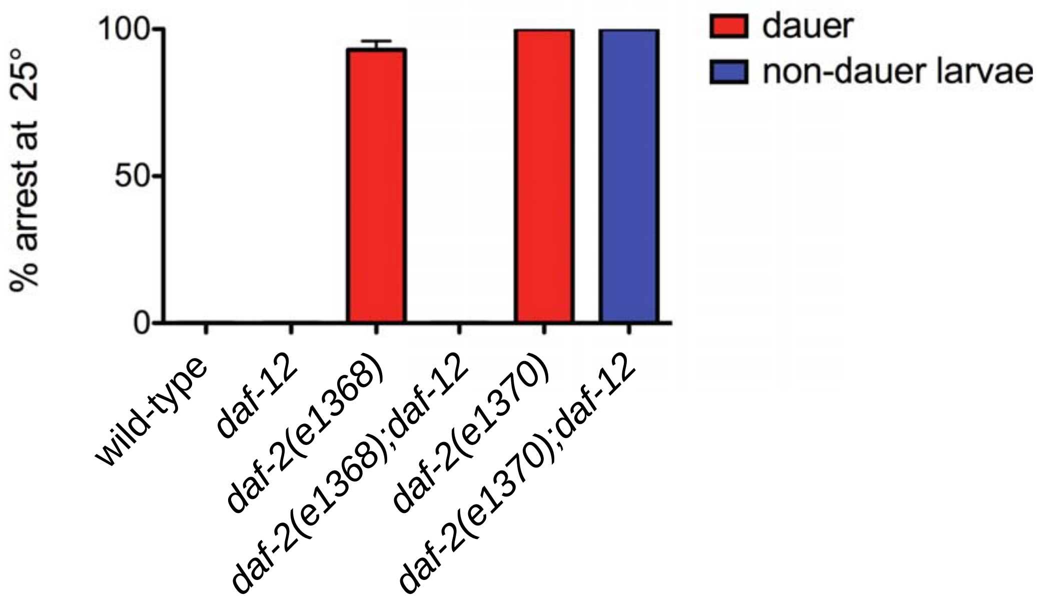

Figure S2 Larval arrest phenotypes of *daf-2;daf-12(null)* double mutants at 25°. *daf-12(null)* suppresses dauer arrest of *daf-2(e1368)* mutant animals [*daf-2(e1368)* v. *daf-2(e1368);daf-12(null), P*< 0.0001]. *daf-2(e1370);daf-12(null)* animals arrest as non-dauer larvae, see text for details. Data represent the average of at least two replicate experiments, with a minimum of 495 animals scored per genotype. Error bars represent SD. All raw data and statistics are presented in Table S2.

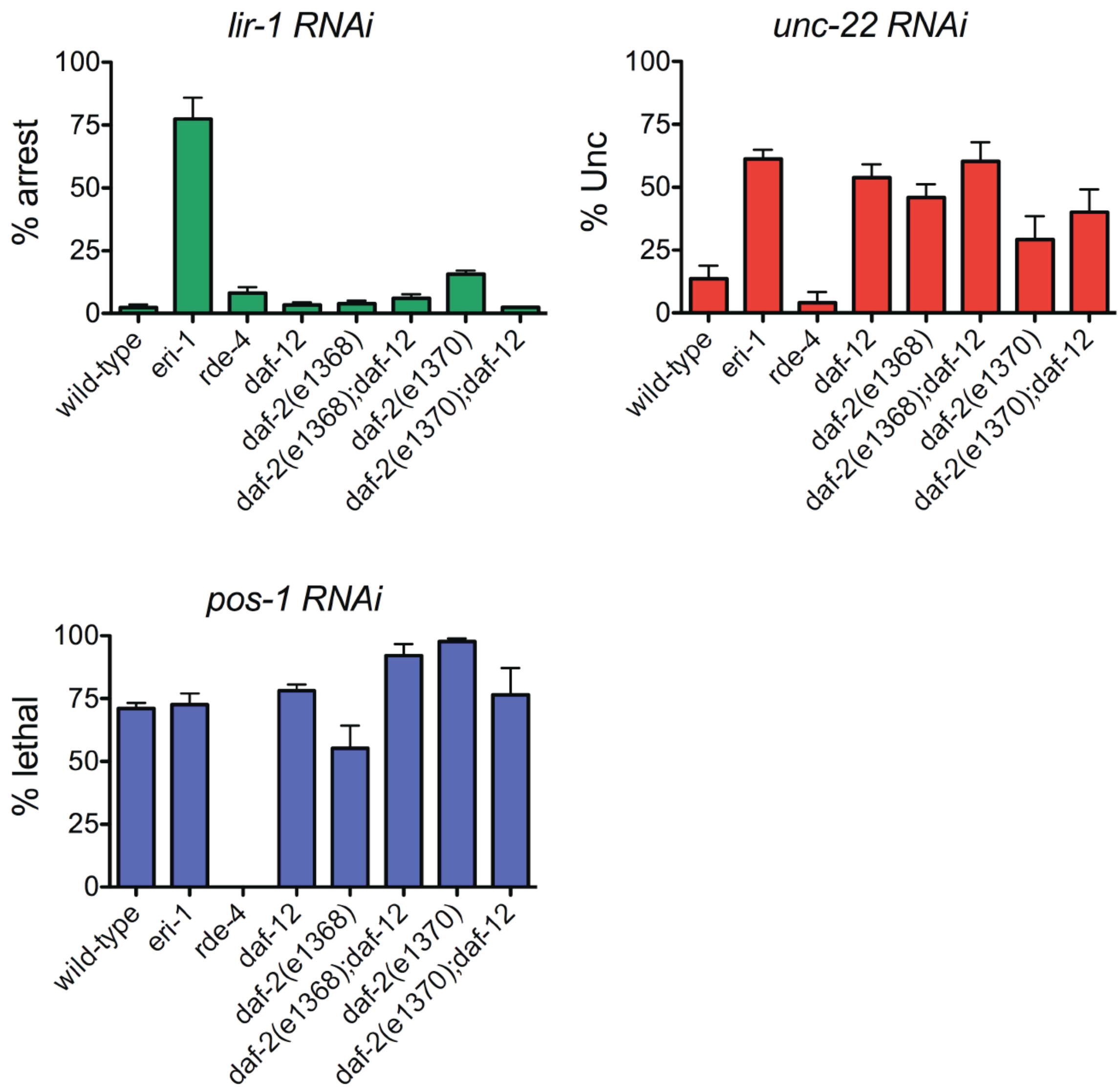

Figure S3 ***daf-12(null)*** **mutation does not cause an RNAi-defective phenotype.** Phenotypes of animals subjected to RNAi of *lir-1*, *unc-22*, and *pos-1* are shown. As controls, the <u>e</u>nhanced-<u>R</u>NA<u>i</u> strain *eri-1(mg366)* and the <u>R</u>NAi-<u>de</u>fective strain *rde-4 (ne301)* are shown. Error bars represent SEM.

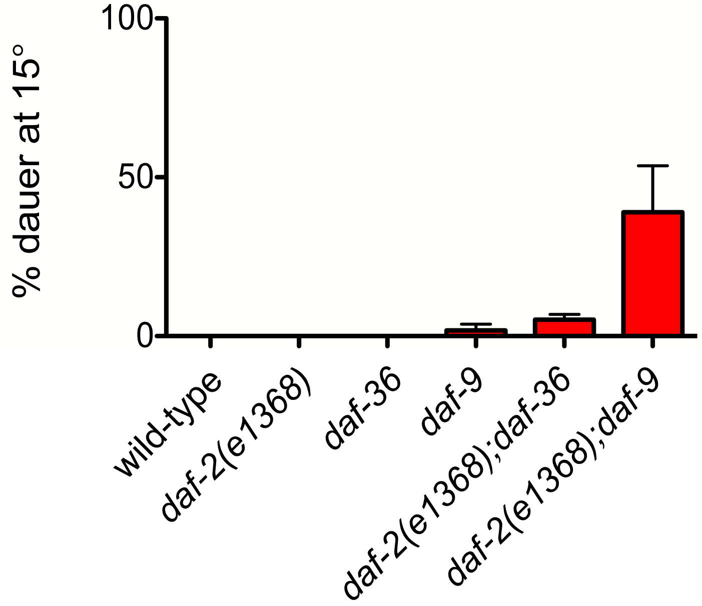

Figure S4 Enhancement of the 15° dauer-constitutive phenotype *daf-2(e1368)* by mutations in genes encoding DA biosynthetic pathway components. *daf-36(null)* and *daf-9(k182)* mutations enhance dauer arrest of animals harboring the Class I *daf-2(e1368)* allele [*daf-2(e1368)* v. *daf-2(e1368);daf-36(null), P* = 0.0388; *daf-2(e1368)* v. *daf-2(e1368);daf-9(k182), P* = 0.0561]. Data represent the average of three replicate experiments, with a minimum of 700 animals scored per genotype. Error bars represent SEM. All raw data and statistics are presented in Table S2.

Table S1 Mutant alleles used in this study.

Gene	Allele	Nature of mutation	Comments	Reference(s)
daf-2	*e1368*	missense, ligand binding domain	Class I	Gems *et al*. 1998; Kimura et al. 1997
daf-2	*e1370*	missense, tyrosine kinase domain	Class II	Gems *et al*. 1998; Kimura et al. 1997
daf-9	*k182*	missense	hypomorphic allele	Gerisch *et al*. 2001
daf-12	*rh61rh411*	nonsense (both mutations)	null; all isoforms affected	Antebi *et al*. 2000
daf-36	*k114*	nonsense	null; Δ^4-, Δ^7-DA not detectable	Rottiers *et al*. 2006; Wollam *et al*. 2011
din-1S	*dh127*	nonsense	null	Ludewig *et al*. 2004
glp-1	*e2141*	missense	animals lack germline when raised at 25°	Priess *et al*. 1987

Table S2 Statistical analysis of all data

Available for download at http://www.g3journal.org/lookup/suppl/doi:10.1534/g3.112.005116/-/DC1
