# Supplementary material for: Influence of Steroid Hormone Signaling on Life Span Control by Caenorhabditis elegans Insulin-Like Signaling
Source: G3 (Bethesda). 2013 May 1;3(5):841–50. doi: 10.1534/g3.112.005116 (PMC3656731; doi:10.1534/g3.112.005116)
Supplement: Supporting Information [file supp_g3.112.005116_FigureS1.pdf]

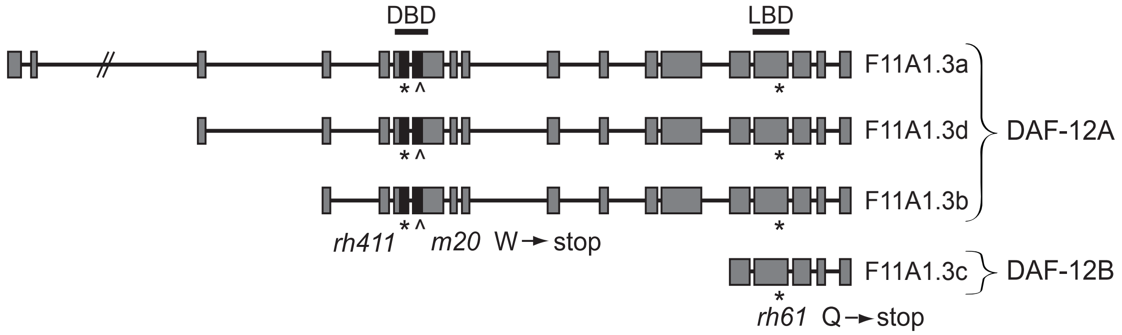

Figure S1 ***daf-12*** **gene structure, transcripts, and relevant mutations.** The structure of *daf-12* transcripts is adapted from WormBase (WBGene00000908). Transcripts corresponding to DAF-12A and DAF-12B isoforms as first described in Antebi *et al.* 2000 and Snow and Larsen 2000 are shown. The location of molecular lesions is taken from Antebi *et al.* 2000 and Snow and Larsen 2000. The *m20* mutation is indicated by a caret, and the *rh411* mutation is indicated by an asterisk in the DNA binding domain. *rh411* is a small deletion/duplication after the first Zn finger in the DNA binding domain that results in an in-frame stop (Antebi *et al.* 2000). Abbreviations: DBD, DNA binding domain; LBD, ligand binding domain.
