# Supplementary material for: Influence of Steroid Hormone Signaling on Life Span Control by Caenorhabditis elegans Insulin-Like Signaling
Source: G3 (Bethesda). 2013 May 1;3(5):841–50. doi: 10.1534/g3.112.005116 (PMC3656731; doi:10.1534/g3.112.005116)
Supplement: Supporting Information [file supp_g3.112.005116_FigureS2.pdf]

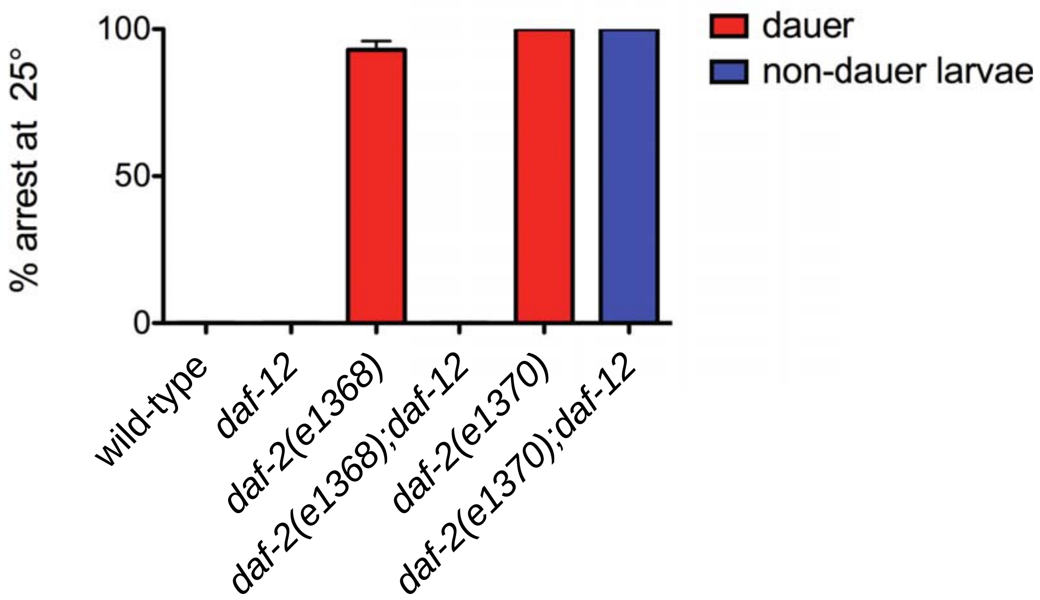


**Figure S2 Larval arrest phenotypes of *daf-2;daf-12(null)* double mutants at 25°.** *daf-12(null)* suppresses dauer arrest of *daf-2(e1368)* mutant animals [*daf-2(e1368)* v. *daf-2(e1368);daf-12(null), P*< 0.0001]. *daf-2(e1370);daf-12(null)* animals arrest as non-dauer larvae, see text for details. Data represent the average of at least two replicate experiments, with a minimum of 495 animals scored per genotype. Error bars represent SD. All raw data and statistics are presented in Table S2.
