# Supplementary material for: Influence of Steroid Hormone Signaling on Life Span Control by Caenorhabditis elegans Insulin-Like Signaling
Source: G3 (Bethesda). 2013 May 1;3(5):841–50. doi: 10.1534/g3.112.005116 (PMC3656731; doi:10.1534/g3.112.005116)
Supplement: Supporting Information [file supp_g3.112.005116_FigureS3.pdf]

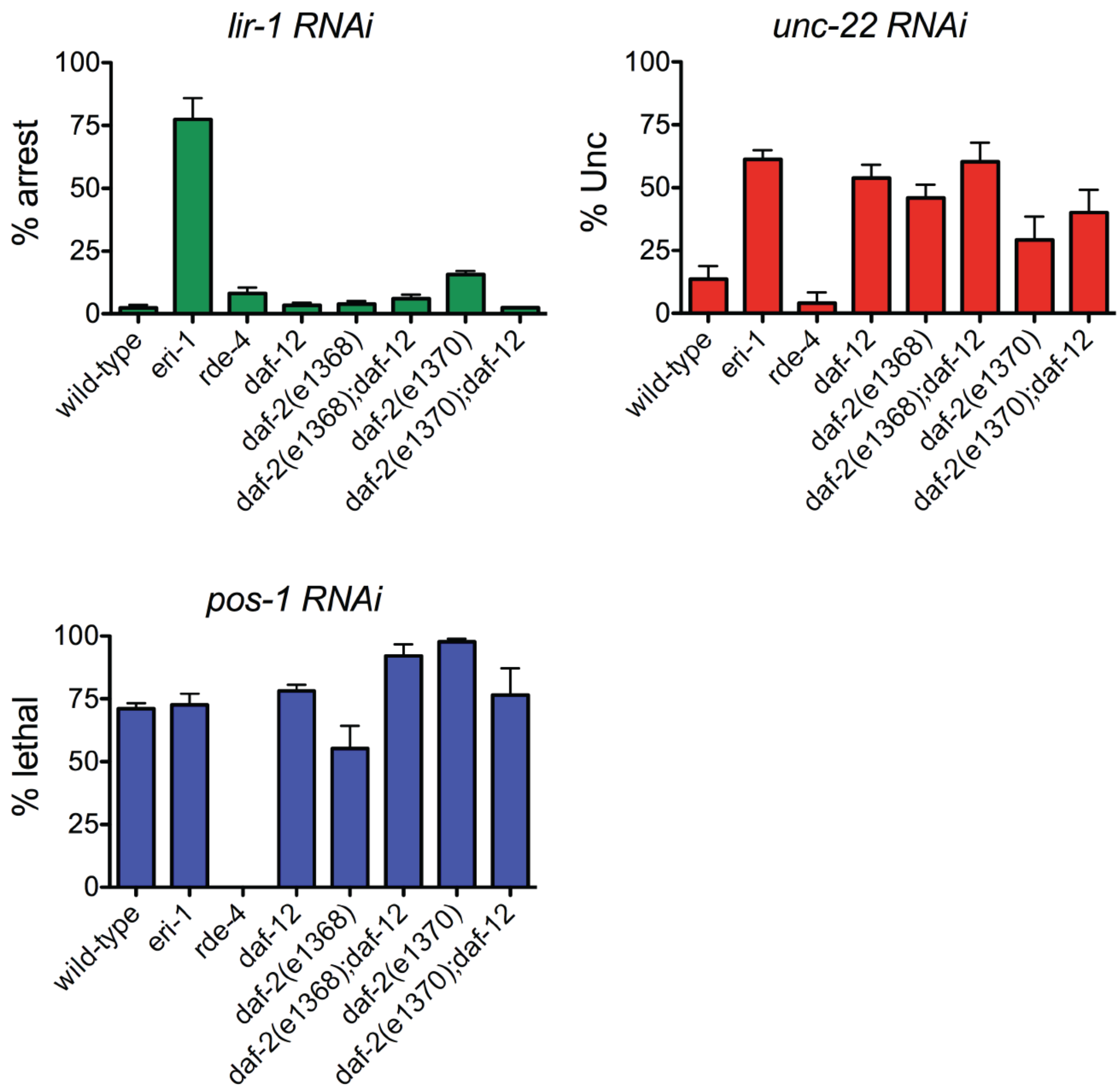

Figure S3 ***daf-12(null)* mutation does not cause an RNAi-defective phenotype.** Phenotypes of animals subjected to RNAi of *lir-1*, *unc-22*, and *pos-1* are shown. As controls, the enhanced-RNA*i* strain *eri-1(mg366)* and the RNAi-defective strain *rde-4 (ne301)* are shown. Error bars represent SEM.
