# Supplementary material for: Influence of Steroid Hormone Signaling on Life Span Control by Caenorhabditis elegans Insulin-Like Signaling
Source: G3 (Bethesda). 2013 May 1;3(5):841–50. doi: 10.1534/g3.112.005116 (PMC3656731; doi:10.1534/g3.112.005116)
Supplement: Supporting Information [file supp_g3.112.005116_FigureS4.pdf]

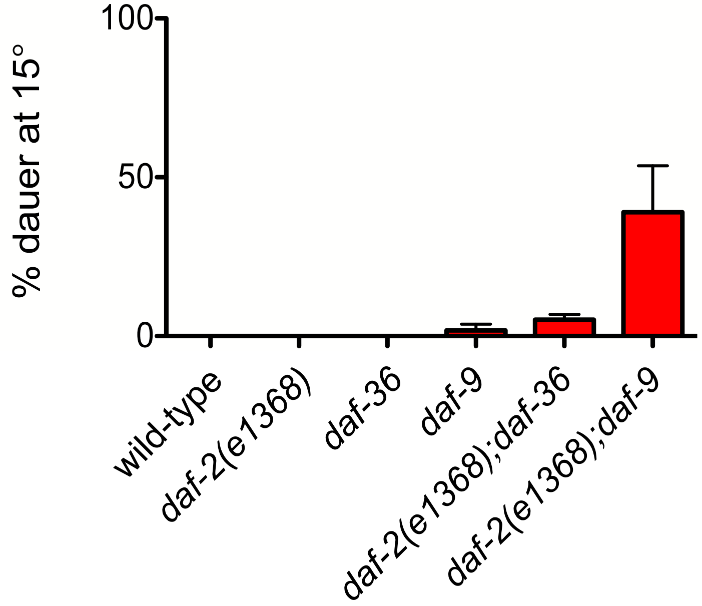

Figure S4 Enhancement of the 15° dauer-constitutive phenotype *daf-2(e1368)* by mutations in genes encoding DA biosynthetic pathway components. *daf-36(null)* and *daf-9(k182)* mutations enhance dauer arrest of animals harboring the Class I *daf-2(e1368)* allele [*daf-2(e1368)* v. *daf-2(e1368);daf-36(null), P* = 0.0388; *daf-2(e1368)* v. *daf-2(e1368);daf-9(k182), P* = 0.0561]. Data represent the average of three replicate experiments, with a minimum of 700 animals scored per genotype. Error bars represent SEM. All raw data and statistics are presented in Table S2.
