# Supplementary material for: Influence of Steroid Hormone Signaling on Life Span Control by Caenorhabditis elegans Insulin-Like Signaling
Source: G3 (Bethesda). 2013 May 1;3(5):841–50. doi: 10.1534/g3.112.005116 (PMC3656731; doi:10.1534/g3.112.005116)
Supplement: Supporting Information [file supp_g3.112.005116_TableS1.pdf]

Table S1 Mutant alleles used in this study.

Gene	Allele	Nature of mutation	Comments	Reference(s)
daf-2	*e1368*	missense, ligand binding domain	Class I	Gems *et al*. 1998; Kimura et al. 1997
daf-2	*e1370*	missense, tyrosine kinase domain	Class II	Gems *et al*. 1998; Kimura et al. 1997
daf-9	*k182*	missense	hypomorphic allele	Gerisch *et al*. 2001
daf-12	*rh61rh411*	nonsense (both mutations)	null; all isoforms affected	Antebi *et al*. 2000
daf-36	*k114*	nonsense	null; Δ^4-, Δ^7-DA not detectable	Rottiers *et al*. 2006; Wollam *et al*. 2011
din-1S	*dh127*	nonsense	null	Ludewig *et al*. 2004
glp-1	*e2141*	missense	animals lack germline when raised at 25°	Priess *et al*. 1987
